# Supplementary material for: OssaNMA: An R package for using information from network meta-analyses to optimize the power and sample allocation of a new two-arm trial
Source: PLoS One. 2023 Dec 21;18(12):e0296020. doi: 10.1371/journal.pone.0296020 (PMC10734972; doi:10.1371/journal.pone.0296020)
Supplement: Attachment — Submitted filename: Response to Reviewers.docx [file pone.0296020.s001.docx]

Dear Dr. Zechen Chong,

Thank you for giving us the opportunity to submit a revised draft of our manuscript. We appreciate the time and effort that you and the reviewers have dedicated to providing your valuable feedback on our manuscript. We have incorporated changes to reflect the reviewers' suggestions and tracked the manuscript's changes. Here is a point-by-point response to the Journal requirements and reviewers’ comments.

Journal Requirements

1- Please ensure that your manuscript meets PLOS ONE's style requirements, including those for file naming.

**Response:** The file names for the figures have been revised to meet the requirements, and other necessary edits have been made to ensure that the manuscript complies with the style requirements.

2 - We note that Figures 4 and 5 in your submission contain copyrighted images. All PLOS content is published under the Creative Commons Attribution License (CC BY 4.0), which means that the manuscript, images, and Supporting Information files will be freely available online, and any third party is permitted to access, download, copy, distribute, and use these materials in any way, even commercially, with proper attribution.

**Response:** We would like to confirm that Figures 4 and 5 do not contain copyrighted images or trademarks. Instead, they are screenshots captured from our personal websites.

3 - Please review your reference list to ensure that it is complete and correct. If you have cited papers that have been retracted, please include the rationale for doing so in the manuscript text, or remove these references and replace them with relevant current references. Any changes to the reference list should be mentioned in the rebuttal letter that accompanies your revised manuscript. If you need to cite a retracted article, indicate the article’s retracted status in the References list and also include a citation and full reference for the retraction notice.

**Response:** We have reviewed our reference list to ensure its completeness and accuracy. We can confirm that none of the papers cited in our manuscript have been retracted.

Comments from Reviewer 1

1- Have the authors made all data underlying the findings in their manuscript fully available?

Reviewer #1: No
Reviewer #2: Yes

**Response:** In the submission process, we provided the Github Link 'https://github.com/fangshuye/NMA-two-arms-sample-size' in the 'Data Availability' section. To ensure the reproducibility of all results presented in our manuscript, we have updated that Github repository to include all datasets and codes. This repository now covers

- sample input data for R shiny app
- dataset and codes for simulation study
- code for generating relevant figures

2 - This method is only tried on simulation analysis to compare the statistical power and standard error with existing network and without existing network. Have the authors ever applied this method on real clinical trial data by any chance to evaluate the power and standard error? I am considering simulation would be overfitting in some circumstances.

**Response:**

In real clinical trial meta-analysis data, the truth (whether there is a difference and how large a difference) would be unknown. Statistical power and standard error can only be assessed under a situation when the truth is known. In a laboratory setting, it might be possible to set up an experiment with truth known. Yet it does not apply to meta-analysis of clinical trials.

We set up a simulation based on real-world data to the extent possible, allowing us to make meaningful comparisons and assess our methodology across a range of realistic scenarios. All simulation parameters are estimated from real data and scenarios are set within a reasonable range of sample sizes and effect sizes.

3 - How did the authors select the existing network? Selecting an accurate and representative network from the existing options can be challengeable, given the potential for inaccuracies in available resources. Were there any specific protocols employed to navigate the incorporation of existing networks into this process?

**Response:** We chose the existing network from a previously published study on interventions for Bovine Respiratory Disease (BRD) in feedlot cattle. This selection was based on several considerations:

- The original paper employed rigorous protocols to ensure the accuracy and representativeness of the existing network.
- We are familiar with the dataset and research questions involved.
- Our motivation for this current paper is to plan future trials related to this specific existing network, making it essential for our simulation study.

[1] O’Connor A, Yuan C, Cullen J, Coetzee J, Da Silva N, Wang C. A mixed treatment meta-analysis of antibiotic treatment options for bovine respiratory disease–an update. Preventive veterinary medicine. 2016;132:130–139

4 - How many existing network studies does it need at minimum to ensure a robust power of the new method? Will the statistical power of existing network meta-analysis affect the statistical power of the two-arm trial and sample size optimization?

**Response:** The number of existing network studies does not directly impact the power of the new method. The key determinant of the new method's power is the standard error of the estimated effect size between treatments A and B from existing Network Meta-Analysis (NMA) (Equation 6). In essence, a single existing study between A and B could suffice if it provides a reliable estimation with a small standard error.

The statistical power of the existing NMA, the statistical power of the two-arm trial analyzed with existing network, and sample size optimization, are directly influenced by the standard error of the estimated effect size from existing NMA. When this standard error is smaller, both the power of existing NMA and the power of the new method (combining new two-arm with existing network) increase, given a fixed total sample size. Alternatively, a smaller total sample size is needed to achieve a predefined level of power.

5 - Providing legends to elucidate your figures, such as in Figure 3, by explaining the significance of nodes and edges, would enhance clarity and comprehension.

**Response**: Thank you for your valuable suggestion. We have now added legends to Figure 3 to provide explanations for the significance of nodes and edges, following PLOS ONE figure guidelines. <https://journals.plos.org/plosone/s/figures#loc-how-to-submit-figures-and-captions>

6 - The clarity of Figure 4 and Figure 5 is compromised due to their low resolution. The figures don’t not show clearly to me. It would be better if you can improve it with higher resolution.

**Response**: We have improved their resolution by reducing the width when taking screenshots, resulting in larger and more legible text within Figures 4 and 5. We trust that this enhancement will provide better visibility.

Comments from Reviewer 2

1. The github page can have more example code shown in README.md

**Response:** Thank you for your suggestion. We have incorporated more example code into the README.md on our GitHub page for our published package (<https://github.com/fangshuye/OssaNMA/tree/master#readme>). For detailed instructions, we also recommend checking the vignettes of our package on CRAN (<https://cran.r-project.org/web/packages/OssaNMA/vignettes/Introduction.html>)

2. In Table 1, simulation power and formula power are quite confusing, and probably need a clearer naming or description in legends.

**Response:** Thank you for your feedback. We have added footnotes under Table 1 to provide explanations for "simulation power," "formula power," "simulation SE," and "formula SE." This clarification should enhance the understanding of the table.

3. In the R package, the two functions are well-explained.

**Response:** We appreciate your positive feedback regarding the clarity and explanation of the two functions in our R package.

We look forward to hearing from you in due time regarding our submission and to responding to any further questions and comments you may have.

Sincerely,

Fangshu Ye, Annette and Chong Wang

Department of Statistics, Iowa State University
